# Facile Fabrication of a Gold Nanocluster-Based Membrane for the Detection of Hydrogen Peroxide

**DOI:** 10.3390/s16071124

**Published:** 2016-07-20

**Authors:** Pu Zhang, Yi Wang, Yibing Yin

**Affiliations:** 1 College of Pharmacy, Chongqing Research Center for Pharmaceutical Engineering, Chongqing Medical University, Chongqing 400016, China; zhangpu51@hotmail.com; 2 Chongqing Key Laboratory of Green Synthesis and Applications, College of Chemistry, Chongqing Normal University, Chongqing 401331, China; ywang@cqnu.edu.cn; 3 Department of Laboratory Medicine, Key Laboratory of Diagnostic Medicine designated by the Ministry of Education, Chongqing Medical University, Chongqing 400016, China

**Keywords:** gold nanoclusters, luminescence, membrane, hydrogen peroxide

## Abstract

In this work, we present a simple and rapid method to synthesize red luminescent gold nanoclusters (AuNCs) with high quantum yield (QY, ~16%), excellent photostability and biocompatibility. Next, we fabricated a solid membrane by loading the as-prepared AuNCs in an agar matrix. Different from nanomaterials dispersed in solution, the AuNCs-based solid membrane has distinct advantages including convenience of transportation, while still maintaining strong red luminescence, and relatively long duration storage without aggregation. Taking hydrogen peroxide (H_2_O_2_) as a typical example, we then employed the AuNCs as a luminescent probe and investigated their sensing performance, either in solution phase or on a solid substrate. The detection of H_2_O_2_ could be achieved in wide concentration ranges over 805 nM–1.61 mM and 161 μM–19.32 mM in solution and on a solid membrane, respectively, with limits of detection (LOD) of 80 nM and 20 μM. Moreover, the AuNCs-based membrane could also be used for visual detection of H_2_O_2_ in the range of 0–3.22 mM. In view of the convenient synthesis route and attractive luminescent properties, the AuNCs-based membrane presented in this work is quite promising for applications such as optical sensing, fluorescent imaging, and photovoltaics.

## 1. Introduction

Fluorescent probes usually play an important role in the assays and imaging applications where fluorescence is employed as a signal. Thus, the systematic design and synthesis of effective fluorescent probes have attracted a lot of attention in the past many years. With the development of nanoscience and nanotechnology, a variety of different luminescent nanomaterials such as semi-conductor quantum dots [1,2], carbon quantum dots [3,4], silicon quantum dots [5], and metal nanoclusters [6,7,8,9] have been synthesized and developed as luminescent probes for a wide range of applications in environmental, food and pharmaceutical analysis, and bioimaging. 

Gold nanoclusters (AuNCs), a member in the family of metal nanoclusters composed of tens to hundreds of Au atoms, are quite different from Au nanoparticles that have characteristic localized surface plasmon resonance (LSPR). AuNCs are new rising stars in the field of nanotechnology, which have recently attracted increasing attention due to their excellent features including subnanometer sizes, high luminescence quantum yield (QY), good biocompatibility and photostability, as well as easy preparation [10]. Moreover, AuNCs with tunable luminescence from the visible to the near-infrared regions can be achieved by manipulating the reaction parameters during the synthesis [11]. Owing to the above advantages, AuNCs have been widely used as novel luminescent probes for the detection of environmental pollutants, proteins (or enzymes), amino acids, and disease-related biomarkers [12,13,14,15,16,17]. For the preparation of AuNCs, there are a lot of strategies, including chemical reduction [18], photochemical reduction [19], phase-transfer [20], chemical etching of Au nanoparticles [21], and transformation from Ag nanoclusters via galvanic replacement reaction [22]. Among these many approaches, chemical reduction in solution phase is the most favorable route for the preparation of AuNCs, in which a variety of different templates such as dendrimers, polyelectrolytes, DNA, proteins, and thiol-containing molecules are available for the synthesis [23,24,25,26,27,28]. 

In this paper, we present a facile and rapid method for the preparation of highly luminescent, photostable, and biocompatible AuNCs in aqueous solution, using bovine serum albumin (BSA) as a template. In addition, based on the strong quenching effect of hydrogen peroxide (H_2_O_2_) on the luminescence of AuNCs, a “turn-off” method for the detection of H_2_O_2_ was established. H_2_O_2_ is involved in many chemical, biological, pharmaceutical, clinical, environmental, and food processes [29]. Of the many analytical techniques that have been reported for the detection of H_2_O_2_, optical (e.g., absorption, and fluorescence/luminescence) and electrochemical (e.g., amperometry) approaches are the most popular [30,31]. Although these methods have practical advantages of sensitivity and/or specificity, they also encounter the problems such as time-consuming or difficult operating procedures, the need for expensive reagents, and/or the presence of serious matrix effects. Thus, rapid, reliable, cost-effective and practical methods for the detection of H_2_O_2_ are in great demand for various applications. Herein, employing the as-prepared AuNCs as a luminescent probe, the detection of H_2_O_2_ in a very wide concentration range of 805 nM–1.61 mM (i.e., across four orders of magnitude) and with high sensitivity (LOD, 80 nM) could be achieved. More importantly, a solid membrane was further fabricated by loading the as-prepared AuNCs in an agar matrix. Compared with the sensing nanomaterials that are usually dispersed in solution, the AuNCs-based solid membrane has advantages of convenience to take along, while still maintaining strong luminescence, and extended duration storage without aggregation, which make it an ideal candidate for sensing applications. In addition, the AuNCs-based membrane could also be used for visual and semi-quantitative detection of H_2_O_2_, using only a portable UV lamp (365 nm) as the detector.

## 2. Materials and Methods 

### 2.1. Chemicals and Materials

Hydrogen tetrachloroaurate (III) hydrate (HAuCl_4_∙3H_2_O) was purchased from Sinopharm Chemical Reagent Co., Ltd. (Shanghai, China). Albumin from bovine serum (BSA) and agar were obtained from Dingguo Changsheng Biotechnology Co., Ltd. (Beijing, China). Hydrogen peroxide (H_2_O_2_) and sodium hydroxide (NaOH) were purchased from Chuandong Chemical Co., Ltd. (Chongqing, China). The Cell Counting Kit-8 (CCK-8) for evaluating the cell growth inhibition of AuNCs was commercially available from the Beyotime Institute of Biotechnology (Jiangsu, China). Milli-Q purified water (18.2 MΩ) was used throughout the experiments.

### 2.2. Instrumentations

Luminescence spectra and photostability of the AuNCs were measured with an F-2500 fluorescence spectrophotometer (Hitachi, Tokyo, Japan). The absolute photoluminescence quantum yield (QY) was obtained by Quantaurus-QY (Hamamatsu, Japan). Atomic force microscopy (AFM) images were measured with a Vecco multimode Nanoscope TM scanning probe microsystem (Vecco, Plainview, NY, USA). X-ray photoelectron spectroscopy (XPS) data were obtained with an ESCALAB 250 X-ray photoelectron spectrometer (Thermo, Waltham, MA, USA). The Fourier transform infrared (FT-IR) spectra were measured with an FTIR-8400S spectrophotometer (Shimadzu, Japan). The photographs of samples were captured with a Canon 6D digital camera (Tokyo, Japan). The cell viability was measured by a Mode 680 Microplate Reader (Bio-Rad, Hercules, CA, USA). A QL-901 vortex mixer (Haimen, China) was employed to mix the solutions.

### 2.3. Synthesis of AuNCs

The BSA-stabilized AuNCs were synthesized according to our previous report with slight modifications [32]. Typically, BSA aqueous solution (1 mL, 50 mg/mL) and HAuCl_4_ solution (1.25 mL, 1.97 mg/mL) were mixed for 5 min under vigorous magnetic stirring. Then, NaOH (1 mol/L) solution was introduced to adjust the pH to 10 (measured with a pH meter), and Milli-Q purified water was added to make up the final volume to 4 mL. This mixture was transferred into a ceramic crucible, covered with a lid (not completely covered up), and allowed to incubate in an oven at 100 °C. The AuNCs product solution (of light brown color) could be obtained after 1 h, which was then diluted with Milli-Q purified water to 4 mL. This product was stored at 4 °C for further use or characterization.

### 2.4. Toxicity Evaluation of AuNCs

The CCK-8 method was used to evaluate the cell viability in the presence of AuNCs (HeLa cells were tested). In detail, the cells were grown in 96-well plates and subsequently incubated with AuNCs at 37 °C for 24 h. The concentrations of the AuNCs (calculated according to the initially introduced Au precursor) were tested in the range of 0–5.8 mM, and each concentration was tested with three sets of parallel samples. After 24 h incubation, each well was washed with PBS buffer for three times. Then, CCK-8 regent was added and incubated with the cells for 1 h at 37 °C. The optical density (OD) was measured at 450 nm with a multi-well plate reader, and the viability of cells could be calculated according to the OD values.

### 2.5. Procedures for the Detection of Hydrogen Peroxide

For the detection in aqueous solution, an appropriate concentration (50 μL, see the main text) of hydrogen peroxide was added to a mixed solution containing 50 μL phosphate buffer (PB, 50 mM, pH 7.0), 50 μL the as-prepared AuNCs (400 nM, calculated according to the initial introduced Au precursor), and 350 μL Milli-Q purified water. After reaction for 15 min at room temperature, the resulting solution was used for spectral measurement and further characterization.

The AuNCs-based membrane was fabricated using agar as a matrix. In detail, 0.1 g agar powder was added to 16 mL boiled Milli-Q purified water and dissolved under stirring firstly. AuNCs (4 mL, 400 nM, calculated according to the initially introduced Au precursor) were then added under stirring when the temperature was decreased to 80 °C. Then, the mixed solution (0.5 mL) was rapidly injected into each well of a 24-well plate. The AuNCs-based membrane could be obtained and peeled off from the 24-well plate once the temperature was decreased to room temperature. For the detection of hydrogen peroxide using the solid membrane, an appropriate concentration (0.2 mL, see the main text) of hydrogen peroxide was injected into the 24-well plate. After reaction for 15 min, the AuNCs-based membrane was peeled off for the measurement of its luminescence.

## 3. Results and Discussion

### 3.1. Synthesis and Optical Properties of AuNCs

In a typical experiment, an aqueous solution of HAuCl_4_ and BSA was mixed under vigorous stirring at room temperature, in which HAuCl_4_ was used as a precursor of Au, and BSA acted as both a reductant and a stabilizer. Subsequently, an appropriate volume of NaOH (1 mol/L) was added to adjust the pH value of the reaction system (pH = 10) to improve the reducing power of BSA and thus accelerate the reduction of Au^3+^ into Au^0^. The reaction solution was then allowed to incubate at 100 °C, and the product, BSA-stabilized AuNCs, could be obtained after 1 h. Compared with the syntheses of BSA-stabilized metal nanoclusters in previously reported works [17,18], the present approach could be achieved in a very short time period (1 h), and the formed AuNCs also had excellent luminescent properties.

**Figure 1:**
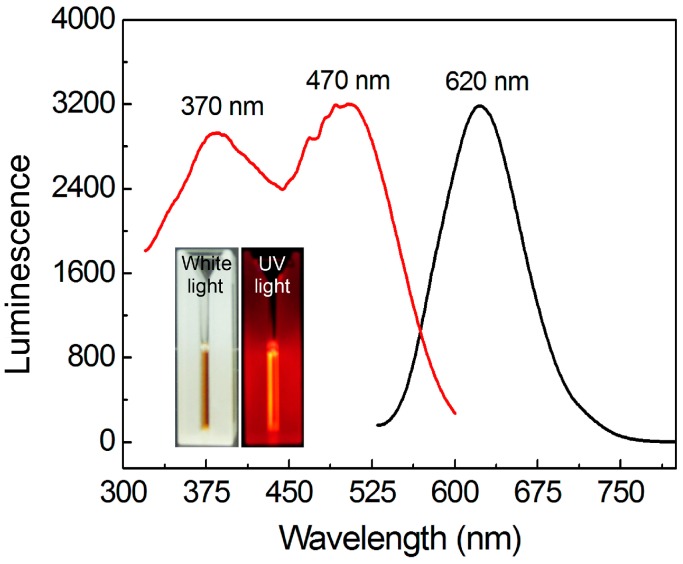
Photoluminescence of the as-prepared AuNCs in the present work. The excitation spectrum shows two peaks at 370 and 470 nm, respectively. The maximal emission peak is located at 620 nm. The inset shows the digital photos of the AuNCs solution under sunlight and 365-nm UV light, respectively.

As shown in Figure 1, a clear light brown color (under sunlight) aqueous solution was obtained, suggesting the as-prepared AuNCs were highly dispersed in solution. Strong red luminescence could be observed when the product was irradiated by a UV lamp (365 nm). The measurement of luminescence spectra shows that the emission peak of the AuNCs is located at 620 nm, which is identical with the observed red emission. Moreover, the strong red luminescence of the as-prepared product could be obtained when it was irradiated either at 370 or 470 nm, because two characteristic peaks were found in its excitation spectrum (measured by fixing the emission wavelength at 620 nm). Further investigations revealed that the absolute photoluminescence QY of such AuNCs could reach as high as 16.2%, which was much higher than the AuNCs prepared in a large number of reported works. Due to the high luminescence QY as well as the large Stokes shift between the maximal excitation and emission peaks, the AuNCs prepared in the present work can act as an ideal optical probe for sensing and imaging.

### 3.2. Structural Characterizations of the AuNCs

**Figure 2:**
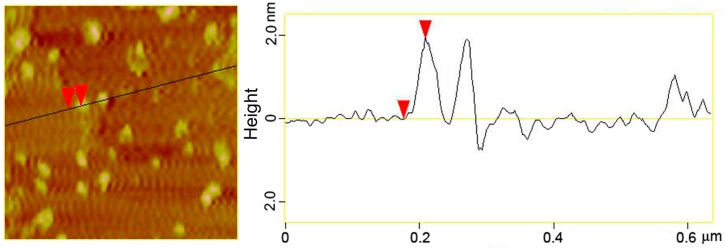
AFM image and the corresponding height measurement of the as-prepared AuNCs.

**Figure 3:**
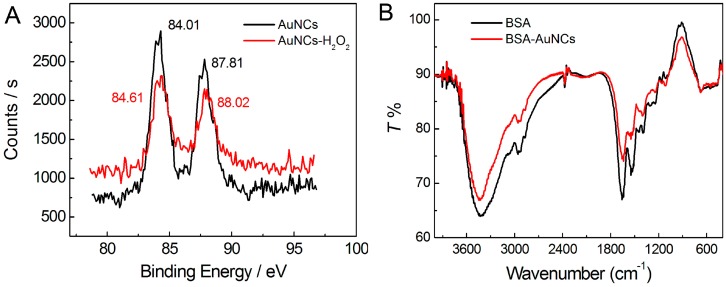
(**A**) XPS spectra showing the binding energy of Au_4f_ before and after the reaction with H_2_O_2_; (**B**) FT-IR spectra of the pure BSA and BSA-stabilized AuNCs, respectively.

To elucidate the size and morphology of the as-prepared AuNCs, AFM imaging was carried out. As shown in Figure 2, the AuNCs are well separated from each other with relatively uniform size. The topographic height measurement further revealed that the size of AuNCs was mostly distributed in the range from 1.0 to 3.5 nm, with an average value of 2.0 nm. Then, XPS and FT-IR were carried out to characterize chemical composition and surface functionalization of the AuNCs. As shown in Figure 3A, the binding energies of 84.01 and 87.81 eV corresponded to the zero-valence Au 4f_7/2_ and Au 4f_5/2_, respectively. This result suggested that Au atoms had been generated through the chemical reduction of Au^3+^ by BSA, and thus aggregated to form small nanoclusters in order to reduce the surface free energy of the whole reaction system. It should be pointed out that BSA was not only a reductant herein, but also a template/stabilizer of the formed AuNCs, which could restrict them in subnanometer size rather than grow into larger Au nanoparticles.

In order to clarify the functions of BSA in the preparation of AuNCs, FT-IR spectra of pure BSA and BSA-stabilized AuNCs were further recorded. As shown in Figure 3B, pure BSA shows a characteristic peak of amide I band (1654 cm^−1^) as expected for a protein with a high proportion of α-helix. The band centered at 1542 cm^−1^ can be attributed to strong primary amine scissoring, and the band at 3430 cm^−1^ can also be attributed to primary amines and associate hydroxyls. The band appearing at 2956 cm^−1^ corresponds to C–H vibration [33]. After the formation of BSA-stabilized AuNCs, no obvious change was observed in the FT-IR spectrum, which suggests that the AuNCs embedded in BSA would not affect the surface-structure of BSA [6].

### 3.3. Effect of pH, Irradiation, and Solvents on the Luminescence of AuNCs

**Figure 4:**
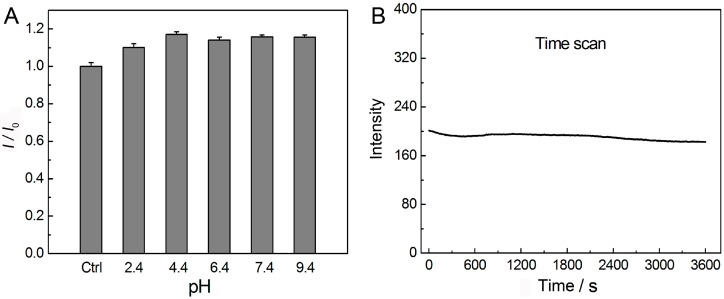
(**A**) Luminescence intensity of the as-prepared AuNCs at different pH. The parameters of *I*_0_ and *I* represent luminescence intensity of the original AuNCs as prepared and after adjusting the pH, respectively; (**B**) Photostability of the AuNCs under continuous irradiation (excitation wavelength: 370 nm) for 1 h.

The as-prepared AuNCs also showed good luminescence stability under different pH conditions in aqueous solution. As shown in Figure 4A, the parameter *I*_0_ represents the luminescence intensity of the original AuNCs as prepared (control group). The parameter *I* represents the luminescence intensity of AuNCs after adjusting the pH value of solution by HCl or NaOH. The results showed that the luminescence of AuNCs was relatively stable in a wide range of pH from 2.4 to 9.4, suggesting the possible optical-related applications of such AuNCs would not be greatly influenced by the pH value of solution. In addition, the photostability of such AuNCs was also investigated, where the sample was excited in a quartzose cell inside a fluorescence spectrophotometer. The excitation wavelength was selected at 370 nm, and the luminescence intensity of 620 nm was recorded as a function of time. As shown in Figure 4B, the luminescence intensity of the AuNCs was always constant, even under the irradiation for 1 h. Thus, compared with organic dyes with photobleaching or semi-conductor quantum dots with photoblink, the as-prepared AuNCs will be more attractive for optical sensing and imaging.

**Figure 5:**
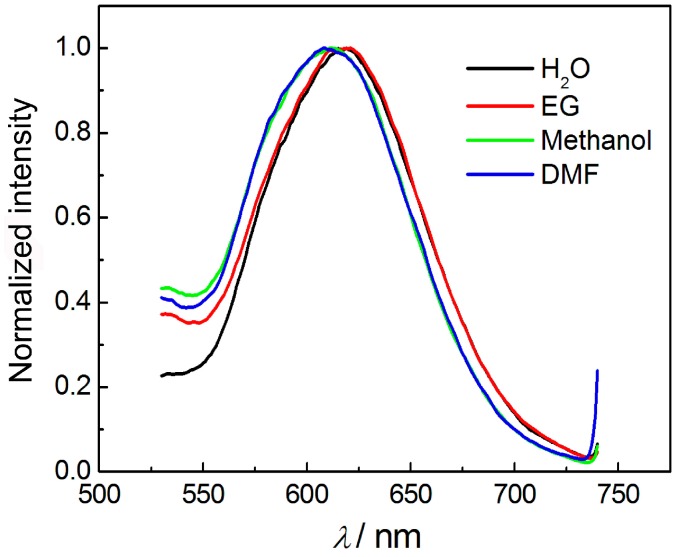
The effect of four different solvents on the luminescence wavelength of AuNCs, including water, ethylene glycol (EG), methanol, and *N,N*-dimethylformamide (DMF).

We further investigated the influence of different solvents on the luminescence of the AuNCs. Figure 5 shows the luminescence spectra of the AuNCs that dispersed in different solvents, including water, methanol, ethylene glycol (EG), and *N*,*N*-dimethylformamide (DMF). It could be observed that the AuNCs dispersed in water and EG showed the maximal emission peak at 620 nm, while the peak blue-shifted for ~10 nm when placed in methanol and DMF. Compared with previous reports on the solvent-dependent luminescence of metal nanoclusters [34], the wavelength shift of the present AuNCs was not remarkable. The slight blue-shift of the luminescence after transferring from water to other solvents might be attributed to the following reasons: (i) solvent-induced aggregation of BSA and thus the BSA-stabilized AuNCs; and (ii) the variation of the microenvironment of the AuNCs.

### 3.4. Toxicity Evaluation of AuNCs in Vitro

**Figure 6:**
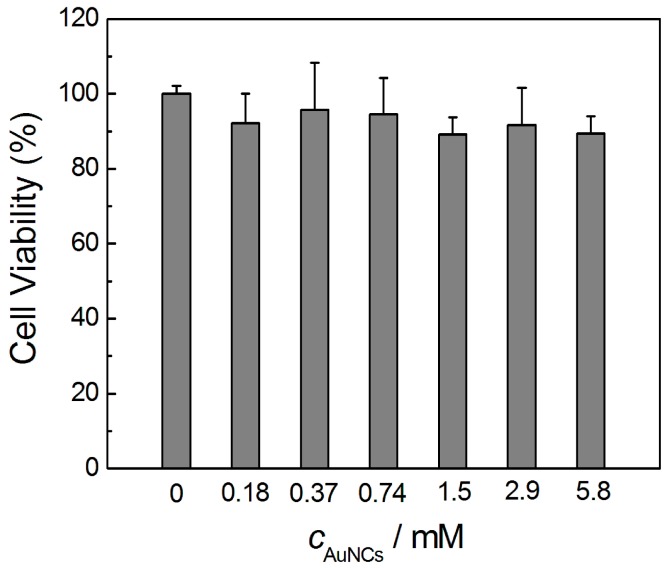
The viabilities of HeLa cells after incubated with different dosages of AuNCs in vitro for 24 h. All the data were collected by conducting three parallel experiments.

To evaluate whether the obtained AuNCs could be applied in biological systems, their toxicological effects against mammalian cells were investigated. In this experiment, HeLa cells were selected, and a concentration range of 0–5.8 mM (also calculated according to the initially introduced Au precursor) of the AuNCs was investigated. Each concentration was tested with three sets of parallel samples. As shown in Figure 6, the viability of HeLa cells was in the range of 89.1%–108.2% when 0–5.8 mM AuNCs were incubated for 24 h, suggesting the excellent biocompatibility of the AuNCs prepared in the present work.

Taking together, we can conclude that the as-prepared AuNCs have high luminescence QY (can reach 16.2%), good optical stabilities under a wide range of pH values and in different solvents, ideal photostability, even under irradiation for relatively long time, excellent biocompatibility, and also can be used or stored in a long period of time. Furthermore, the surfaces of AuNCs can be easily modified through chemical reaction owing to the rich functional groups (e.g., amino and carboxyl groups) in BSA template, making them ideal luminescent probes for sensing and biological imaging.

### 3.5. Detection of Hydrogen Peroxide in Solution Phase

Using H_2_O_2_ as a typical target, we further demonstrated the sensing performances of the as-prepared AuNCs. The quantificational detection of H_2_O_2_ is important since it is usually involved in a large number of chemical, biological, clinical, environmental and food processes. Firstly, we studied the interaction mechanism between the AuNCs and H_2_O_2_ by XPS. 

**Figure 7:**
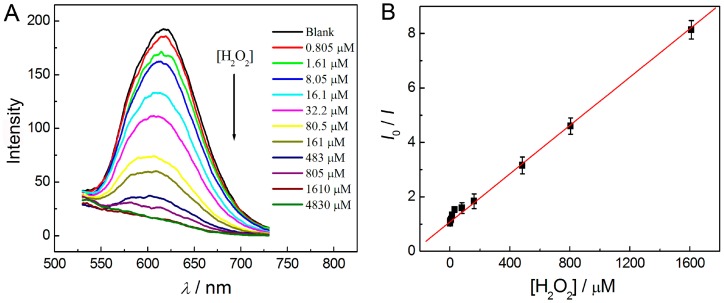
Quantificational detection of H_2_O_2_ in solution phase using the as-prepared AuNCs as a luminescent probe: (**A**) luminescence quenching of the AuNCs with the addition of different amounts of H_2_O_2_ (the concentrations of H_2_O_2_ are 0–4830 μM from top to bottom) in solution; (**B**) the values of *I*_0_/*I* as a function of the concentration of H_2_O_2_ in the range of 805 nM–1.61 mM in solution.

As shown in Figure 3A, the binding energies corresponding to the zero-valence Au 4f_7/2_ (84.01 eV) and Au 4f_5/2_ (87.81 eV) shifted to 84.61 and 88.02 eV, respectively. This result suggested that part of the zero-valence Au atoms had been transformed into Au^+^ through oxidation by H_2_O_2_ [35,36,37]. Thus, the luminescence of AuNCs could be strongly quenched by the interaction between AuNCs and H_2_O_2_. Under the optimal conditions, the as-prepared BSA-stabilized AuNCs (40 μM) were used for the detection of H_2_O_2_ in PB buffer (50 mM, pH 7.0) at room temperature. As shown in Figure 7, the luminescence intensity of the AuNCs gradually decreased with increasing the concentration of H_2_O_2_. The values of *I*_0_/*I* increased linearly (*R*^2^ = 0.99) with increasing concentration of H_2_O_2_ over the range of 805 nM–1.61 mM. The limit of detection (LOD) for H_2_O_2_ was 80 nM, based on a signal-to-noise ratio (S/N) of 3. Compared with most other reported methods for the detection of H_2_O_2_ using nanomaterials as probes [38,39,40,41,42,43,44,45], our method shows the advantages of a very wide detection range (across four orders of magnitude) as well as high sensitivity.

### 3.6. Detection of Hydrogen Peroxide on Solid Membrane

Nanomaterials often encounter the problem of aggregation in solution phase due to their high surface energy, which usually influences their sensing performance. Moreover, nanoparticles dispersed in solution are inconvenient to take along and store, which also limits their practical applications. Although the BSA-stabilized AuNCs prepared in this work were very stable in aqueous solution, we further loaded the AuNCs in an agar matrix and thus developed a more convenient and stable solid membrane as the optical probe for the detection of H_2_O_2_. Specifically, 80 nM AuNCs and 5% (*w*/*v*) agar were mixed at a relatively high temperature (typically, 80 °C), and injected into a 24-well plate (0.5 mL of the mixed solution for each well). The solid membrane could be obtained and peeled off from the 24-well plate once the temperature had decreased to room temperature.

**Figure 8:**
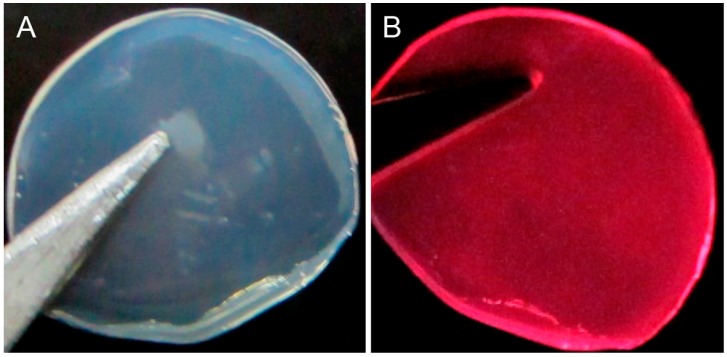
Photographs showing the as-prepared AuNCs-involved membrane, which can be used as a solid probe for the detection of H_2_O_2_: (**A**) under sunlight; (**B**) under UV light (365 nm).

**Figure 9:**
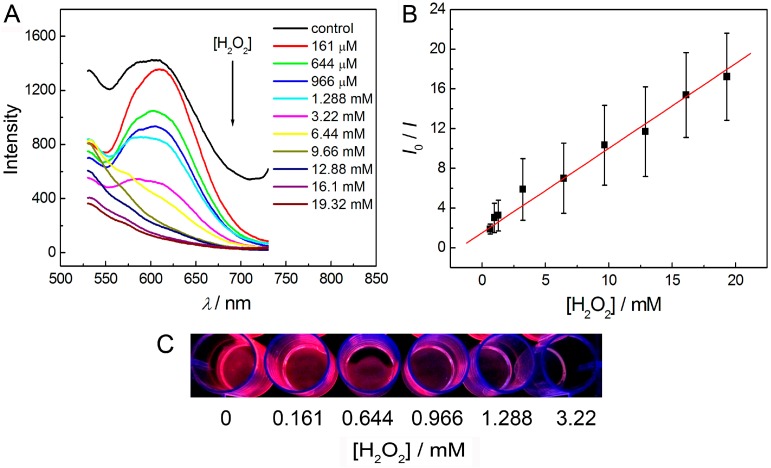
Detection of H_2_O_2_ using AuNCs-based solid membrane: (**A**) luminescence quenching of the AuNCs with the addition of different amounts of H_2_O_2_ (the concentrations of H_2_O_2_ are 0–19.32 mM from top to bottom) on AuNCs-based membrane; (**B**) the values of *I*_0_/*I* as a function of the concentration of H_2_O_2_; (**C**) a photograph showing the visual detection of H_2_O_2_ in the concentration range of 0–3.22 mM.

As shown in Figure 8, a semitransparent solid membrane could be obtained after it was peeled off from the 24-well plate. This membrane also exhibited strong red luminescence under a 365 nm UV light. This result demonstrated that an AuNCs-based membrane which could also be applied in the quantificational detection of H_2_O_2_ could be successfully fabricated using agar as a matrix. As shown in Figure 9, the luminescence intensity of the AuNCs-based membrane gradually decreased with the increase of the concentration of H_2_O_2_. The values of *I*_0_/*I* increased linearly (*R*^2^ = 0.99) upon increasing the concentration of H_2_O_2_ over the range of 161 μM–19.32 mM. Moreover, it could be observed by eyes that the red luminescence of the solid membrane was gradually decreased when the concentration of H_2_O_2_ increased in the range of 0–3.22 mM under the irradiation by a portable UV lamp (365 nm). It means that the AuNCs-based membrane can be an ideal candidate for visual and semi-quantitative detection of H_2_O_2_.

## 4. Conclusions

In conclusion, we have presented a facile method for the preparation of highly luminescent, photostable, and biocompatible AuNCs. Owing to the oxidation of Au atoms into Au (I) by H_2_O_2_, the luminescence of AuNCs could be greatly quenched after the interaction with H_2_O_2_. Thus, an analytical method for H_2_O_2_ determination was further established based on the relationship between the luminescence quenching and the concentration of H_2_O_2_. The detection range could be from 805 nM to 1.61 mM, and the LOD for H_2_O_2_ could reach as low as 80 nM in solution phase. Moreover, we further anchored the AuNCs into the agar matrix and fabricated a solid membrane with strong red luminescence and improved stability. Such an AuNCs-based membrane has been successfully used for the detection of H_2_O_2_ over the concentration range of 161 μM–19.32 mM, and also has achieved the visual detection of H_2_O_2_ in the range of 0–3.22 mM using a portable UV lamp. In view of the convenient synthesis route and attractive luminescent properties, the as-prepared AuNCs-based solid membrane in the present work will be quite promising in applications such as visual sensing, optical imaging, and photovoltaics.
